# Characterization and Antioxidant Activity of Essential Oil of Four Sympatric Orchid Species

**DOI:** 10.3390/molecules24213878

**Published:** 2019-10-28

**Authors:** Francesco Saverio Robustelli della Cuna, Jacopo Calevo, Elia Bari, Annalisa Giovannini, Cinzia Boselli, Aldo Tava

**Affiliations:** 1 CREA Research Centre for Animal Production and Aquaculture, Viale Piacenza 29, 26900 Lodi, Italy; aldo.tava@crea.gov.it; 2 DDS Department of Drug Sciences, University of Pavia, Viale Taramelli 12, 27100 Pavia, Italy; elia.bari@unipv.it (E.B.); cinzia.boselli@unipv.it (C.B.); 3 CREA Research Centre for Vegetable and Ornamental Crops, Corso degli Inglesi 508, 18038 Sanremo (IM), Italy; jacopo.calevo@unito.it (J.C.); annalisa.giovannini@crea.gov.it (A.G.); 4 DBIOS Department of Life Sciences and Systems Biology, University of Torino, Viale Mattioli 25, 10125 Torino, Italy

**Keywords:** *Anacamptis coriophora*, *Anacamptis pyramidalis*, *Ophrys holosericea*, *Serapias vomeracea*, alkenes, volatiles, pollinators

## Abstract

The volatile fractions from fresh inflorescences of naturally growing orchids *Anacamptis coriophora* (L.) R. M. Bateman, Pridgeon & M. W. Chase *subsp. fragrans* (Pollini), *Anacamptis pyramidalis* (L.) R. *Ophrys holosericea* (Burm.) Greuter and *Serapias vomeracea* (Burm. f.) B. were isolated by steam distillation and analyzed by GC/FID and GC/MS. Saturated hydrocarbons were quantified as the major constituents of the volatile fraction (47.87–81.57% of the total essential oil), of which long-chain monounsaturated hydrocarbons accounted from 9.20% to 32.04% of the total essential oil. Double bond position in linear alkenes was highlighted by dimethyl disulfide derivatization and MS fragmentation. Aldehydes (from 3.45 to 18.18% of the total essential oil), alcohols (from 0.19% to 13.48%), terpenes (from 0.98 to 2.50%) and acids (0.30 to 2.57%) were also detected. These volatiles compounds may represent a particular feature of these plant species, playing a critical role in the interaction with pollinators. DPPH assay evaluating the antioxidant activity of the essential oils was carried out, showing a dose-dependent antioxidant activity.

## 1. Introduction

Pollination of flowers by animals is often influenced by a wide variety of volatile molecules [1,2]. The floral scent in plants has the primary aim to attract and guide pollinators [3,4], playing a critical role both in long- and short-distance attraction [2,5,6]. In fact, bees learn odours easier and more rapidly than colours [3,7]. Furthermore, the floral scent may thus influence/drive pollinator constancy [3,8], which ensures pollen transfer, reduces pollen loss and contributes to maintaining both the plant reproductive fitness and their barriers among species [9,10]. Additional functions of floral volatile chemicals occur as defensive and protective mechanisms vs. biotic and abiotic stresses [11,12,13]. This may explain the wide variety of volatiles fragrances emitted by orchids acting as key characters to drive pollinators when food or sexual deception takes place [2].

Orchidaceae is considered one of the well-represented flowering plant families, worldwide distributed accounting approximately 28,000 species [14]. This abundance leads to a great complexity of floral scents; in fact, orchids can potentially produce almost all the fragrances occurring in nature [15]. This wide variety of floral scents is primarily due to the combination of the great number of orchid species and of the evolution of pollination systems. The mechanisms of deception in orchids include generalized food deception, food-deceptive floral mimicry, brood-site imitation, shelter imitation, pseudo-antagonist, rendezvous attraction and sexual deception, where generalized food deception is the most common mechanism (38 genera) followed by sexual deception (18 genera) [16,17]. In the Orchidoideae subfamily, in particular, consisting of 7 subtribes and about 3630 species [18], the sexual deception mechanism can be recognized for orchid genera such as *Drakea* or *Ophrys* [18]. Furthermore, orchids using food-deceptive strategy show flowers resembling those of rewarding co-occurring species [19].

Italy hosts about 236 orchid species [20], that frequently co-occur in the same habitat and population [21]. A peculiar pollination strategy is the sexual deception of the genus *Ophrys*: in this orchid genus the shape of the labellum looks like the female abdomen of the bee pollinator species and the floral scent contains interactive chemicals resembling the sex pheromones of pollinators. These intriguing visual and olfactory signals are, therefore, of critical importance to driving pollinator choices [22]. On the other hand, genera such as *Orchis*, *Dactylorhiza* and some *Anacamptis* employ a generalized food-deceptive strategy [19], in which these flowers provide floral cues indicating a *food* reward while animal pollination is achieved without providing nectar, pollen or other food rewards. On the contrary species of the genus *Serapias* employ an unusual pollination strategy called shelter deception. In fact, these flowers, nectarless and not so brightly coloured, form a small tube used by pollinators as a refuge during cold or rainy weather to rest or sleep [23,24,25].

The aim of the present investigation was to isolate and compare the essential oils from inflorescences of four sympatric orchid species (*Anacamptis coriophora* (L.) R.M. Bateman, Pridgeon & M. W. Chase *subsp. fragrans* (Pollini), *Anacamptis pyramidalis* (L.) R., *Serapias vomeracea* (Burm. f.) B. and *Ophrys holosericea* (Burm) Greuter), co-occurring in the same natural site in Italy, in order to characterize their chemical composition and antioxidant activity.

## 2. Results

The essential oil obtained by steam distillation from fresh inflorescences were evaluated as 1.3 mg for *A. coriophora subsp. fragrans*, 1.8 mg for *A. pyramidalis*, 2.6 mg *O. holosericea* and 3.4 mg for *S. vomeracea*, respectively. The yields were evaluated as 0.03%, 0.02%, 0.52% and 0.10% (weight/fresh weight basis), respectively. Table 1 shows the results of qualitative and quantitative essential oil analyses on the Elite-5 MS column. The compounds are listed in order of their elution time and reported as percentages of the total essential oil. The total number of peaks for *A. coriophora subsp. fragrans* was 60 with number of identified peak of 43 (72% identification), *A. pyramidalis* was 58 with number of identified peak of 45 (78% identification). *O. holosericea* was 59 with number of identified peak of 49 (83% identification), *S. vomeracea* was 65 with number of identified peak of 59 (91% identification). As evidenced, the main represented volatiles constituents are saturated hydrocarbons, especially in *A. coriophora subsp. fragrans* followed by *S. vomeracea*, *O. holosericea* and *A. pyramidalis*, and unsaturated hydrocarbons mainly present in *O. holosericea* essential oil. Differences in the qualitative and quantitative composition of the volatile essential oils obtained from the four sympatric Italian orchids have been observed.

### 2.1. Anacamptis Coriophora subsp. Fragrans

Major constituents of the volatile fractions of this orchid species were found to be saturated hydrocarbons (81.57% of the total essential oil), from which heneicosane (25.10%), nonadecane (20.51%), tricosane (17.16%), pentacosane (9.31%) and heptacosane (3.43%) are the most abundant compounds. A series of unsaturated linear chain hydrocarbons were identified, of which 9-pentacosene and 9-heptacosene represent the 3.39% and 2.30% of the total volatiles, followed by 9-tricosene and 1-hexadecene accounting for 1.42% and 1.21% of the total essential oil, respectively. Aldehydes are present in the percentage of 3.45%, being nonanal (1.61%), phenylacetaldehyde (0.70%) and anisaldehyde (0.68%) the most represented. Alcohols (0.19%) and terpenes (0.98%) consisted of 2,5-dimethoxybenzyl alcohol (0.19%), thymol (0.36%) and α-copaene (0.26%), respectively.

### 2.2. Anacamptis Pyramidalis

Major constituents of the volatile fractions of *A. pyramidalis* were found to be saturated hydrocarbons accounting for 52.43% of the total essential oil. Tricosane (17.17%), pentacosane (16.24%), heneicosane (7.50%) and heptacosane (6.04%) are the most abundant constituents of this class of compounds. Aldehydes present as 16.19% were basically represented by nonanal (5.44%), heptanal (4.02%) phenylacetaldehyde (3.82%) and octadecanal (1.44%). Alcohols (13.48%) consist of 2-phenylethanol (12.11%) followed by benzyl alcohol (1.13%). A series of unsaturated linear chain hydrocarbons (10.34%) was instead identified with 9-pentacosene and 9-heptacosene as the 4.69% and 3.90%, followed by 7-heptacosene evaluated as 1.38% of the total essential oil. Acids are also detected as 2.57%, being heptanoic acid (1.41%) the most abundant followed by nonanoic acid (1.16%). Terpenes (2.50%) are mainly represented by α-copaene (0.45%), thymol (0.30%) and α-cadinene (0.14%).

### 2.3. Ophrys Holosericea

Saturated hydrocarbons, accounting for 47.87% of the total essential oil were found to be the major constituents also of *O. holosericea* volatiles. Tricosane (27.71%), pentacosane (6.84%), heneicosane (4.34%), heptacosane (2.22%) are the most abundant compounds. A series of unsaturated linear chain hydrocarbons (32.04%) was also identified, of which 7-pentacosene (16.60%) was the major represented, followed by 9-tricosene (3.19%), 9-pentacosene (2.95%), 7-tricosene (2.72%), 7-heptacosene (2.36%), 9-heptacosene (2.17%) and 11-pentacosene (1.77%). Aldehydes (10.74% of the total essential oil) consist mainly of nonanal (4.65%), phenylacetaldehyde (2.07%), heptanal (1.39%) and octadecanal (1.00%). Alcohols (3.20% of the total) are represented by benzyl alcohol (2.59%) and terpenes (1.74% of the total) by thymol (0.66%), α-copaene (0.56%) and γ-muurolene (0.48%).

### 2.4. Serapias Vomeracea

More than 50% of the *S. vomeracea* volatile fraction consists of saturated hydrocarbons accounting for 53.29% of the total essential oil. Pentacosane (17.59%), tricosane (14.21%), heneicosane (5.68%), heptacosane (4.99%), nonadecane (2.45%), tetracosane (1.90%) and tetradecane (1.68%) were the most representative compounds. Monounsaturated linear chain hydrocarbons (18.63%) were also identified, of which the 9- and 7- isomers were the most represented. In details the rank order is: 9-pentacosene (3.92%) > 7-heptacosene (3.71%) > 9-heptacosene (2.87%) > 7-pentacosene (2.48%) > 9-tricosene (1.19%) > 7-tricosene (1.18%). Aldehydes accounting for 18.18% of the total volatiles consist mainly of nonanal (7.87%), phenylacetaldehyde (3.91%), heptanal (1.57%), undecanal (1.35%) and octadecanal (0.93%). Acids (1.92% of the total) are represented by palmitic acid (0.77%), nonanoic acid (0.71%) and heptanoic acid (0.37%). Terpenes (2.44%) account for *trans*-β-farnesene (0.84%), γ-muurolene (0.39%) and thymol (0.28%). The only alcohol found in *S. vomeracea* volatile fraction was diacetone alcohol as 1.03% of the total essential oil.

### 2.5. Venn’s Diagram

**Figure 1:**
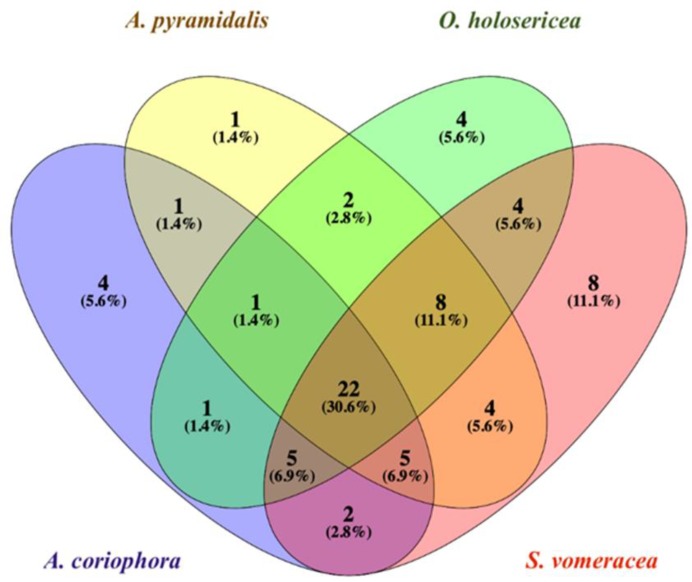
Venn’s diagram shows both the number of compounds shared and unshared/peculiar among the four orchid species. Percentages are referred to the total number of compounds found, not to the relative abundance [27].

Figure 1 shows the Venn’s diagram [27] in which 22 compounds are shared among all the 4 sympatric Italian orchids. Even if just a few, some peculiarities were found to be species-specific. In detail, four compounds were found only in *A. coriophora subsp. fragrans*, i.e., creosol, anisaldehyde, γ-eudesmol and methyl-p-methoxycinnammate, although poorly represented (<1% of the total essential oil). In *A. pyramidalis* only one compound, 2-phenylethanol (12.11% of the total essential oil), seems to be species-specific, further characterizing this species. *O. holosericea* showed to have four peculiar compounds, i.e., heptanol, 3.5-octadien-2-one, 2.3-dimethyldecane and 11-pentacosene. It should be stressed that only the latter one is present with a valuable amount, reaching 1.77% of the total essential oil. On the contrary, *S. vomeracea* was the species showing eight unique chemicals, being *trans*-β-farnesene, that represents the 50% of total terpenes, palmitic acid and 9-heneicosene the most abundant reaching the 0.84%, 0.77% and 0.71% of the total essential oil, respectively.

### 2.6. DPPH Assay

**Figure 2:**
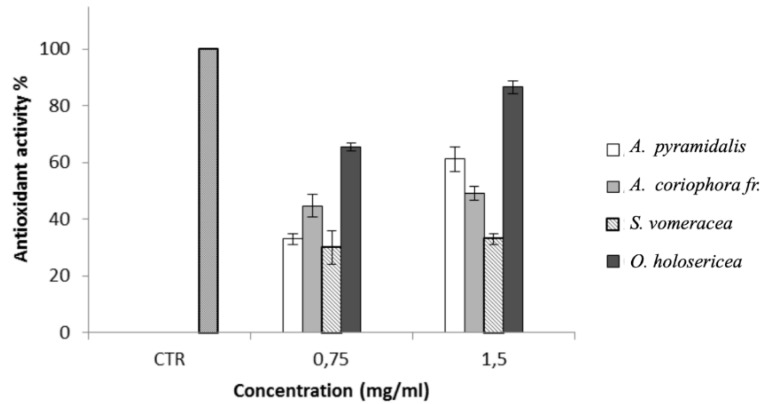
ROS-scavenging activity (%) of the tested extracts.

All samples demonstrated a good, dose-dependent, antioxidant activity by the DPPH assay (Figure 2). ANOVA analysis evidenced that the ROS-scavenging activity is strongly influenced both by the sample and concentrations tested (*p* < 0.0001). In particular, *O. holosericea* shows the strongest antioxidant activity, especially at a concentration of 1.5 mg/mL. For *S. vomeracea*, the concentration did not influence the antioxidant activity (*p* > 0.05).

## 3. Discussion

A high percentage of saturated hydrocarbons has been detected in the volatile fraction of all the four Italian sympatric orchid species. The presence of saturated hydrocarbons in higher percentage with respect to other published research [28] is probably due to the different extraction procedures like solid-phase microextraction that work at lower extraction temperatures. In detail, a series of homologous linear chain compounds ranged from C_9_ to C_29_ have been reported for all the terrestrial and epiphytic orchids. The presence of hydrocarbons as allelochemicals is associated with epicuticular wax chemistry playing an important role in plant/herbivore interactions. Saturated alkanes exerted an interesting, although limited, activity in pollinator deception in different floral species [25,29,30,31].

The position of the double bond in linear alkene isomers was determined by GC/MS after iodine-catalyzed reaction with dimethyl disulfide. Derivatization procedure was applied to alkene mixtures, which have chemotaxonomic value for the pollinator populations. Linear chain monounsaturated hydrocarbons were previously identified in several orchid species with a high content of these compounds in flowers [30]. Although these compounds seem not to act are as specific contributors to the aroma of the plant, they might be crucial in modulating plant-herbivore interaction [32]. In fact, several studies demonstrated that this class of compounds is endowed with an interesting, although limited, activity in pollinator deception in Orchidaceae [21,31]. Furthermore, multifactorial mechanisms involved in protection actions from environmental factors such as water loss, thermal- or UV-related stress, seem to be related to the occurrence of hydrocarbons [30,32].

Another class of substances that might be involved in plant-insect interaction is reported to be benzenoids [33,34]. *A. pyramidalis*, a specialized species and mainly dependent on butterflies for pollination [35,36,37,38], shows a strong presence of 2-phenylethanol (12.11% of the total volatiles) in its flower’s scent compared to the other orchis species (see Table 1). It should be stressed that benzenoids play a critical role in pollinator attraction strategy of *A. pyramidalis*, as well as those of two other terrestrial orchids, *Nigritella nigra* (today accepted as *Gymnadenia nigra*) (L) Reichb. f. (2-phenylethanol) [15,39] and *Gymnadenia conopsea* (L) Br.R., (benzyl acetate) [15]. Given the volatile extract’s composition, which included ROS-scavenging compounds, the antioxidant activity for each extract has been evaluated by DPPH method. All samples demonstrated a good antioxidant activity that is probably related to the presence of benzyl alcohol (*A. pyramidalis* 1.13%, *O. holosericea* 2.59%) and phenylacetaldehyde (*A. coriophora subsp. fragrans* 0.70%, *A. pyramidalis* 3.82%, *O. holosericea* 2.07% and *S. vomeracea* 3.91%) as previously reported for essential oils from *Laurus nobilis* and *Fagopyrum* species [40,41], to the presence of thymol (*A. coriophora subsp. fragrans* 0.36%, *A. pyramidalis* 0.30%, *O. holosericea* 0.66% and *S. vomeracea* 0.28%) and α-copaene (*A. coriophora subsp. fragrans* 0.26%, *A. pyramidalis* 0.45% and *O. holosericea* 0.56%) as reported for essential oil from *Cinnamodendron dinisii* and *Siparuna guianensis* [42,43]. However, even if benzenoids have been observed to be predominant in specialist butterfly-pollinated flower scents, these compounds have been also found in generalist plants, suggesting that they might be emitted by both specialists and generalists [44]. It should be stressed that differences in floral scents, visual attraction and reward systems are decisive for chemical communication in pollination strategies of sympatric orchids to guarantee pollination efficiency and fidelity.

In detail, *Ophrys* flowers act as “false female” mimicking her visual, tactile and olfactory stimuli, so male pollinators attempt to copulate with the orchid labellum removing and delivering pollen, a process termed pseudocopulation. It should be noticed that the complex blend of different odours mimicking female pheromones mainly consists of long-chain alkanes and alkenes derivatives and that the relative alkanes and alkenes abundance makes each floral scent unique resulting in different pollinator species attraction [22,30].

As regards *S. vomeracea*, its flowers form a small tube, which pollinators use basically as nest-replacement or refugee during rainy weather, although the pollination strategy of this orchid relies not only in the floral shape but also in the olfactory attractors to assure sufficient degree of pollinator fidelity; in fact both visual and olfactory signals are of critical importance in pollinator choice. Again, according to the literature [45] alkanes and alkenes have been found to be very important volatile components in this orchid species scent as reported in Table 1. In fact, a solitary bee such as *Megachile rotundata* has been found to mark its nest with an olfactory trace consisting of a mixture of alkanes and alkenes similar to that present in the *Serapias* and *Ophrys* scents [30].

Another class of substances that might be involved in plant-insect interaction is reported to be benzenoids [33,34]. *A. pyramidalis*, a specialized species and mainly dependent on butterflies for pollination [35,36,37,38], shows a strong presence of 2-phenylethanol in its flower’s scent as reported (see Table 1). It should be stressed that benzenoids play a critical role in pollinator attraction strategy of *A. pyramidalis*, as well as those of two other terrestrial orchids, *Nigritella nigra* (today accepted as *Gymnadenia nigra*) (L) Reichb. f. (2-phenylethanol) [15,39] and *Gymnadenia conopsea* (L) Br.R., (benzyl acetate) [15].

However, even if benzenoids, in this case mainly 2-phenylethanol, have been observed to be predominant in specialist butterfly-pollinated flower scents, these compounds have been also found in generalist plants, suggesting that they might be emitted by both specialists and generalists [44].

Among the species investigated *A. coriophora subsp. fragrans* can be occasionally pollinated by Lepidoptera (even if they are not the main pollinators), as shown in Table 2 [46]. 

Unlike *A. pyramidalis*, in *A. coriophora subsp. fragrans* alkanes and alkenes are the main constituents of the scent. The results reported in the present paper are different from those reported in the scarce literature available data [47,48,49]. In this investigation, we analyzed pre-pollinated flowers of *A. coriophora subsp. fragrans* chemotype *europaeus*, while both inflorescences and mature seeds of *A. coriophora subsp. fragrans* chemotype *africanus* [47], or inflorescences of *A. coriophora subsp. coriophora* [48], respectively, were used by other Authors.

In conclusion keeping in mind that these four orchids colonize the same environment, bloom in the same time and share also some pollinators, the data reported in the present paper strongly suggest that each species may attain a peculiar combination of olfactory, tactile and/or visual floral signs suitable to explain different interactive communication systems between plants and pollinators. Furthermore, it is reasonable to assess that each orchid species is able to gain especially through different signals a sufficient level of pollinator fidelity and thus maintain its genetic identity even in a population of admixed orchid species

## 4. Materials and Methods

### 4.1. Plant Material

Inflorescences of the four orchid species were collected in May 2016 in Pompeiana (Imperia, Italy, 438,547 N 78,909 E) according to the regional law and with the legal permission of Regional Authorities (Region Liguria, Prot. n. PG/2016/104503). Plants were identified according to Chase and colleagues [48]. A type specimen for each species is deposited in the living collection of CREA-OF (Sanremo, Italy) with the accession numbers ANcf01, ANpy01, OPho01, and SEvo01 for *A. coriophora subsp. fragrans*, *A. pyramidalis*, *O. holosericea* and *S. vomeracea*, respectively. The flowers were cut and immediately placed in a pyrex bottle containing 100 mL of methylene chloride as a preservative agent and stored at −20 °C.

### 4.2. Isolation of Volatile Fraction

Flowers of *A. coriophora subsp. fragrans* (4.6 dried g), *A. pyramidalis* (9.3 dried g), *O. holosericea* (0.5 dried g) and *S. vomeracea* (3.3 dried g), to which ethyl decanoate was added as internal standard, were steam distilled together with methylene chloride in a Clevenger-type apparatus for 1 h. The distillate, saturated with NaCl, was extracted with freshly distilled diethyl ether (3 × 100 mL), dried over anhydrous Na_2_SO_4_ concentrated at first with a rotary evaporator and finally using a gentle stream of N_2_ and then analyzed by GC/FID and GC/MS.

### 4.3. Fractionation and Alkylthiolation of Alkenes

A portion of the essential oil from each sample was placed onto a glass column (7 × 30 mm) of silica gel 60, 230–400 mesh (Merck, Milano, Italy), preconditioned with pentane [29]. The non-polar fraction was eluted with 2 mL of pentane and used for the determination of double bond position in alkenes by alkylthiolation according to reported method [50].

### 4.4. GC/FID Analysis

The analyses were carried out using a Hewlett Packard model 5890 GC, equipped with Elite-5MS (5% phenyl methyl polysiloxane, Supelco, Sigma Aldrich, Milano, Italy) capillary column of (30 m × 0.32 mm i.d.) and film 0.32 μm thick. The carrier gas was He at a flow of 1 mL/min. One μL aliquots of each essential oil were manually injected in “split” mode (30:1). The oven temperature program included an initial isotherm of 40 °C for 5 min, followed by a temperature ramp to 260 °C at 4 °C/min, and a final isotherm at this temperature for 10 min. Injector and detector temperatures were set at 250 and 280 °C, respectively.

### 4.5. GC/MS Analysis

The analyses were carried out using a GC Model 6890 N, coupled to a benchtop MS Agilent 5973 Network, equipped with the same capillary column and following the same chromatographic conditions used for the GC/FID analyses. The carrier gas was He at a constant flow of 1.0 mL/min. The essential oils were diluted prior to analysis (1mg/10mL in *n*-hexane), and 1.0 µl of the diluted solution was manually injected into the GC system with a split ratio of 30:1. The ion source temperature was set at 200 °C, while the transfer line was at 300 °C. The acquisition range was 40–500 amu in electron-impact (EI) positive ionization mode using an ionization voltage of 70 eV.

### 4.6. Identification and Quantification of the Essential Oil Components

The identification of the essential oil volatile components was performed by their retention indices (RI) and their mass spectra, and by comparison with a NIST 98 and Wiley 5 MS libraries, as well as with literature data [26]. Retention indices were calculated by an Elite-5MS capillary column using a series of n-alkanes (C_8_-C_23_) under the same GC conditions as for samples [51]. The relative amount of each individual component of the essential oil was expressed as percent peak area relative to the total peak area from GC/FID analyses of the whole extract.

### 4.7. DPPH Assay

The ROS-scavenging activity of the essential oils was evaluated by the DPPH (2,2-diphenyl-2 picrylhydrazyl hydrate) method according to the previously described method with slight modifications [52,53,54]. At first, the essential oils were solubilized in dimethyl sulfoxide and then diluted in methanol at final concentrations of 1.5 and 0.75 mg/mL. 270 μL of DPPH (0.028% *w*/*v* in methanol) was mixed with 30 μL of each sample. Reaction mixtures were incubated in the dark for 20 min at room temperature before measuring the absorbance at 517 nm using a microplate reader (Synergy HT, BioTek, Swindon, United Kingdom). Ascorbic acid (1.25 mg/mL) was used as a positive control, while the reaction mixture without any sample was used as a negative control. ROS scavenging activity percentage was calculated as follows:

% activity = (A − B)/A × 100(1)

where A is the absorbance of the negative control and B is the absorbance of the tested sample. Analyses were performed in three replicates.

### 4.8. Statistical Analysis

Results of DPPH assay are reported as mean ± standard deviation for the values with a normal distribution (or interquartile range and median for the values that did not adhere to the Gaussian distribution). For the data with normal distribution, an analysis of variance (ANOVA) was performed, considering the compound and its concentration as fixed factors, while the inhibition percentage as the dependent variable. The significance criterion was set to *p* < 0.05.

## Figures and Tables

**Table 1:** Percentage composition of the volatile fraction from inflorescences of *A. coriophora subsp. fragrans*, *A. pyramidalis*, *O. holosericea* and *S. vomeracea*.

			Percentage Peak Area			
Compound	RI ^a^	RI ^b^	*A. coriophora subsp. Fragrans*	*A. pyramidalis*	*O. holosericea*	*S. vomeracea*
Hexanal	801	799	tr	0.30	tr	0.21
2,4-Dimethyl heptane	820	819	-	0.13	0.21	0.19
2-Methyl-2-pentenal	821	829	-	0.16	0.30	0.24
Diacetone alcohol	841	844	-	0.24	0.37	1.03
Heptanal	901	901	0.17	4.02	1.39	1.57
Benzaldehyde	961	957	0.10	0.59	0.18	0.13
Heptanol	972	971	tr	tr	0.10	tr
Nonane	1000	999	0.45	-	0.08	0.06
Octanal	1001	1003	-	-	0.07	0.22
Benzyl alcohol	1032	1033	tr	1.13	2.59	tr
Phenylacetaldehyde	1042	1042	0.70	3.82	2.07	3.91
2,4-Dimethyldecane	1067	1068	0.56	0.55	1.24	0.57
3,5-Octadien-2-one	1072	1071	-	-	0.08	-
Heptanoic acid	1083	1083	0.14	1.41	-	0.37
Undecane	1100	1100	-	0.31	-	0.18
Nonanal	1102	1104	1.61	5.44	4.65	7.87
2-Phenylethanol	1107	1112	-	12.11	tr	tr
p-Cresol	1158	1158	0.87	-	-	-
Octanoic acid	1173	1173	-	-	-	0.07
1-Dodecene	1192	1192	-	-	-	0.19
Dodecane	1200	1200	0.47	0.42	0.45	0.26
Decanal	1204	1206	-	0.17	0.15	0.49
Anisaldehyde	1251	1253	0.68	-	-	-
Nonanoic acid	1275	1272	0.16	1.16	tr	0.71
Thymol	1277	1281	0.36	0.30	0.66	0.28
Undecanal	1307	1307	-	tr	0.36	1.35
2,5-Dimethoxy benzyl alcohol	1328	1327	0.19	-	0.14	-
α-Copaene	1376	1376	0.26	0.45	0.56	tr
1-Tetradecene	1390	1392	-	tr	-	0.39
Tetradecane	1400	1400	0.45	0.54	0.50	1.68
Dodecanal	1412	1409	-	0.05	0.26	0.38
*trans*-Caryophyllene	1418	1421	-	0.09	0.04	-
*trans*-β-Farnesene	1452	1458	-	-	-	0.84
γ-Muurolene	1471	1468	0.08	0.05	0.48	0.39
Pentadecane	1500	1500	0.15	0.19	0.12	0.36
α-Cadinene	1537	1538	0.16	0.14	-	0.09
1-Hexadecene	1591	1592	1.21	-	-	0.51
Hexadecane	1600	1600	0.19	0.14	0.19	0.15
Tetradecanal	1613	1613	-	0.09	0.31	0.60
γ-Eudesmol	1627	1635	0.12	-	-	-
Methyl-p-methoxycinnamate	1617	1614	0.58	-	-	-
Heptadecane	1700	1700	0.24	0.24	0.45	0.65
Pentadecanal	1713	1713	-	0.11	-	0.28
1-Heptadecene	1755	1748	0.06	-	-	0.40
Octadecane	1800	1800	-	-	-	0.08
Nonadecane	1900	1900	20.51	2.26	0.56	2.45
Isophytol	1944	1949	-	1.47	tr	0.84
Palmitic acid	1950	1960	-	-	-	0.77
Eicosane	2000	1999	1.56	0.14	-	0.17
Octadecanal	2021	2020	0.19	1.44	1.00	0.93
9-Heneicosene	2071	2073	-	-	-	0.71
Heneicosane	2100	2100	25.10	7.50	4.34	5.68
Docosane	2200	2199	0.96	-	1.01	0.80
11-Tricosene	2261	2265	0.17	-	0.15	0.65
9-Tricosene	2279	2274	1.42	-	3.19	1.19
7-Tricosene	2287	2280	-	-	2.72	1.18
Tricosane	2300	2300	17.16	17.17	27.71	14.21
Tetracosane	2400	2400	0.76	-	1.58	1.90
11-Pentacosene	2469	2469	-	-	1.77	-
9-Pentacosene	2474	2474	3.39	4.69	2.95	3.92
7-Pentacosene	2483	2482	-	tr	16.60	2.48
Pentacosane	2500	2500	9.31	16.24	6.84	17.59
Hexacosane	2600	2600	0.27	0.47	-	0.85
9-Heptacosene	2676	2675	2.30	3.90	2.17	2.87
7-Heptacosene	2683	2681	0.34	1.38	2.36	3.71
Heptacosane	2700	2699	3.43	6.04	2.22	4.99
9-Nonacosene	2876	2874	0.31	0.37	0.13	0.43
Nonacosane	2900	2902	-	0.09	0.37	0.47
Saturated hydrocarbons			81.57	52.43	47.87	53.29
Unsaturated hydrocarbons			9.20	10.34	32.04	18.63
Aldehydes			3.45	16.19	10.74	18.18
Alcohols			0.19	13.48	3.20	1.03
Terpenes			0.98	2.50	1.74	2.44
Acids			0.30	2.57	tr	1.92
Miscellaneous			1.45	-	0.08	-

RI ^a^: Retention Indices from literature [26]. RI ^b^: Retention Indices calculated by GC/FID using n-alkane series (from C_8_ to C_23_) under the same analytical conditions as for samples. tr, traces (<0.01%); >0.01% quoted to nearest 0.01%.

**Table 2:** Relationship between each sympatric Italian orchid and its pollinators, according to GIROS [46].

Species	Lepidotpera	Diptera	Hymenoptera	Coleoptera
*Anacamptis coriophora subsp. Fragans*	Nymphalidae Zygaenidae	Tachinidae Bombyliidae	Apidae Halictidae Vespidae	Oedemeridae
*Anacamptis pyramidal*	Arctiidae Crambidae Hesperidae Lycaenidae Nymphalidae Noctuidae	Bombyliidae Conopidae Empididae	Apidae	Oedemeridae
*Ophrys holosericea*	-	-	Apidae Formicidae	Rutelidae
*Serapias vomeracea*	-	-	Apidae Crabronidae Eumenidae Halictidae Megachilidae	Scarabaeidae
